# A migratory herbivore responds to rapid changes in food supply: diet and body condition of an arctic caribou herd at onset of green-up

**DOI:** 10.1093/conphys/coag073

**Published:** 2026-09-28

**Authors:** Cecelia J Giesen, Joelle Hepler, Shawna A Karpovich, Christopher S Swingley, Perry S Barboza

**Affiliations:** Department of Ecology and Conservation Biology, Texas A&M University, Wildlife, Fisheries, and Ecological Sciences Building, 534 John Kimbrough Blvd, College Station, TX 77843, USA; Department of Wildlife Conservation, Department of Wildlife Conservation, Alaska Department of Fish & Game, 1300 College Rd, Fairbanks, AK 99701, USA; Department of Wildlife Conservation, Department of Wildlife Conservation, Alaska Department of Fish & Game, 1300 College Rd, Fairbanks, AK 99701, USA; ABR, Inc.—Environmental Research & Services, 2842 Goldstream Road, Fairbanks, AK 99709, USA; Department of Ecology and Conservation Biology, Texas A&M University, Wildlife, Fisheries, and Ecological Sciences Building, 534 John Kimbrough Blvd, College Station, TX 77843, USA

**Keywords:** Forage quality, functional response, isotopes, phenological progression*, Rangifer tarandus granti*

## Abstract

Annual migration of ungulates is often correlated with regional and seasonal plant growth, but are smaller scale movements of individuals related to fitness? We investigated the response of wild caribou to variation in plant phenology. We used faecal transects to evaluate spatial differences in diet in one growing season after the onset of spring plant growth had occurred (green-up). We captured 110 females (1–15 years) from the Teshekpuk Caribou herd over three years (14–28 June 2022–2024). We measured diet and corresponding indices of body condition of captured caribou at various stages of green-up. Plant growth (normalized differentiation vegetation index) at capture locations was greatest in 2023 when captures followed the onset of green-up. We sampled faeces and blood to measure concentrations of albumin and urea N in serum, and to measure δ^15^N and δ^13^C to track N and C from diet. Faecal concentrations of N (crude protein) indicated that diet quality was highest in 2023 (20.3%) after onset of green-up when caribou were closest to the coast and lowest in 2022 (5.6%) before onset of green-up when caribou were furthest inland. Serum albumin (4.4 g/dL) and urea N (9.9 mmol/L) concentrations were greatest in 2023, which was consistent with high N supplies after onset of green-up. Faecal δ^15^N values were lowest in 2022 and highest in 2024, which suggests diets dominated by lichens and graminoids, respectively. In 2023, even though some samples were from later into green-up, intermediate values for δ^15^N and low values for δ^13^C in blood and faeces were consistent with browsing near the coast. Changes in diet that can influence gain of body protein and thus the growth and reproductive potential of females were related to locations of caribou in the 14d window of green-up.

## Abbreviations

AIC, Akaike information criteria
ASIF, Alaska Stable Isotope Facility
C, Carbon
BCG, Bromocresol green
DTM, Digital terrain model
DOY, Day of year
GEBCO, General Bathymetric Chart of the Oceans
GPS, Global positioning system
IFSAR, Interferometric synthetic aperture radar
N, Nitrogen
NDVI, Normalized differentiation vegetation index
OLS, Ordinary least squares
PET, Polyethylene terephthalate
RCF, Relative centrifugal force
TCH, Teshekpuk caribou herd
VHF, Very high frequency

## Introduction

Ungulates provide ecosystem services including sustaining plant communities and promoting biodiversity (Hobbs, 1996; Augustine and McNaughton, 1998) while also provisioning human communities (Naranjo *et al.*, 2004; Borish *et al.*, 2022). Migrating ungulates extend these services to various plant, animal and human communities across ecoregions and political boundaries. Ungulate migrations have been associated with food, water, terrain, weather, predators, pests and humans (Wolfe *et al.*, 2000; Aikens *et al.*, 2017; Leclerc *et al.*, 2019; Abrahms *et al.*, 2021; Atmeh *et al.*, 2024). However, studies have highlighted the importance of resource tracking as a foundational element of migration for ungulates in which movements correlate with patterns of plant growth (Mysterud *et al.*, 2001; Hebblewhite *et al.*, 2008; Monteith *et al.*, 2014; Aikens *et al.*, 2017; Abrahms *et al.*, 2021).

Resource tracking of herbivores has been described in relation to patterns of plant growth (i.e. green-up) by the forage maturation (Fryxell, 1990) and green wave hypotheses (Drent *et al.*, 1978). These hypotheses provide the framework for why and how herbivores move in relation to phenological progression to maximize their access to feeding on the highest quality forage (Drent *et al.*, 1978; Fryxell, 1990; Aikens *et al.*, 2017). Studies using GPS collars have correlated animal movements and plant phenology using satellite derived metrics such as the normalized difference vegetation index (NDVI) (Pettorelli *et al.*, 2011; Johnson *et al.*, 2018). Though it has long been inferred that resource tracking along migratory routes will affect individuals and therefore population dynamics, current research has lacked the fine scale physiological data to support it. Seasonal physiological responses have been well studied, including metabolic rates (Parker *et al.*, 2009; Arnold, 2020), reproductive investments (Barboza and Parker, 2008; Taillon *et al.*, 2013) and daily activity patterns (Arnold *et al.*, 2018; Barboza *et al.*, 2024). Large-scale benefits of resource tracking have been documented in elk (*Cervus elaphus*) (Proffitt *et al.*, 2016; Middleton *et al.*, 2018) and mule deer (*Odocoileus hemionus*) over the scale of a season (Ortega, 2023). Finer scale physiological responses to shifts in phenology (e.g. spring green-up) have not been investigated in large ungulates.

Generally, ungulates migrate to areas with consistent patterns of forage availability to maximize foraging returns while also reducing risks to fitness from terrain, weather, predators, pests and people (Monteith *et al.*, 2018; Kauffman *et al.*, 2021). Barren-ground caribou (*Rangifer tarandus granti*) migrate as far as 1200 km each year (Joly *et al.*, 2019) and must contend with a short window of plant growth when food quality and quantity provide sufficient energy, nitrogen (N), and minerals for growth and reproduction (Barboza *et al.*, 2018; Oster *et al.*, 2018). Migratory caribou move to calving grounds where the availability and duration of forage is likely to provide consistent returns (Bischof *et al.*, 2012; Cameron *et al.*, 2020). Consequently, migratory caribou and other arctic ungulates use photoperiod and other environmental cues to modulate daily activity and reproductive cycles (Arnold *et al.*, 2018). The strong pulse of food availability at the start of plant growth in spring would be expected to stimulate a pronounced response in body condition and thus physiological evidence for actively following resources among years.

Seasonal and annual changes in body condition of ungulates have been evaluated with body mass (Taillon *et al.*, 2011), body size (Buntgen *et al.*, 2020), and metrics of lean mass and fat mass (Cook *et al.*, 2021). Body mass has been used in conjunction with morphological measurements of the limbs, thorax and the jaw to account for differences in frame size (Taillon *et al.*, 2011). More acute responses in body condition rely on blood parameters to track mobilization of energy and protein from body stores, or net uptake from the diet into tissues (Barboza and Parker, 2006; Soppela *et al.*, 2008; Johnson *et al.*, 2010; Daleo *et al.*, 2025). Serum albumin, urea N and creatinine have been used as indicators of changes in body protein balance (Franzmann, 1985).

Albumin, urea N and creatinine are associated with changes in live mass rather than subcutaneous fat on the rump of reindeer (*R. tarandus*) over winter, indicating a response to loss of lean mass (Milner *et al.*, 2003). Serum concentrations of albumin reflect changes in integration of amino acid supply from the diet and recirculation of amino acids in the body by the liver (Barboza *et al.*, 2009; Parker *et al.*, 2009). Albumin concentrations can therefore decline when dietary supplies of N fall below the requirement (Soppela *et al.*, 2008; Parker *et al.*, 2009).

Likewise, urea N also reflects supplies of amino acids to the liver from diet and body tissue (Barboza *et al.*, 2009; Parker *et al.*, 2009). Urea N therefore increases when amino acids are oxidized from either the diet or the body stores (Soppela *et al.*, 2008; Parker *et al.*, 2009). Before green-up, high urea N reflects mobilization of body protein whereas after green-up, high urea N is associated with increasing N intake (Barboza *et al.*, 2009; Parker *et al.*, 2009). Creatinine provides an indicator of renal clearance that qualifies changes in urea N (Barboza *et al.*, 2009; Parker *et al.*, 2009). High urea N accompanied by elevated creatinine indicates low clearance from the kidneys rather than an increase in urea N production (Barboza *et al.*, 2009; Parker *et al.*, 2009). Conversely, increases in urea N at low concentrations of creatinine reflect changes in urea formation from either the diet or body stores (Barboza *et al.*, 2009; Parker *et al.*, 2009).

Stable isotopes can be used to track dietary and endogenous sources of C and N (Gustine *et al.*, 2014b). Isotopic changes in serum protein track changes in diet over 10 days whereas urea responds to fasting and diet within 2 days in reindeer and caribou (Barboza and Parker, 2008; Barboza *et al.*, 2020; Meier *et al.*, 2024). Total N and its stable isotope δ^15^N in faecal and blood fractions may also indicate diet quality, plant functional groups and body protein dynamics (Gustine *et al.*, 2012; Gustine *et al.*, 2014a). Isotopic pools of C and N in body tissues are a mixture of dietary input (exogenous substrates) and the material in the body (endogenous pools). Fibre particles probably reflect diet ingested within the last 4–5 days, which is the mean retention time for reindeer consuming browse in summer (Singer *et al.*, 2024). In comparison, blood cells typically mature over ~100 days with an isotopic signature similar to lean mass gained prior to green-up in female caribou and reindeer (Barboza and Parker, 2008; Barboza *et al.*, 2020). Serum proteins reflect diet and metabolism over the previous 10–25 days (Prinsen and de Sain-van der Velden, 2004; Levitt and Levitt, 2016).

We investigated the response of wild caribou to variation in plant phenology using two hypotheses: (i) We predict the diet of caribou will vary to reflect their geographical location within the calving grounds, and (ii) we predict body condition will respond to phenological changes in diet quality. To test hypothesis 1, we collected faecal samples in mid-July within the calving grounds of the Teshekpuk Caribou Herd (TCH). Sampling occurred on the eastern and western sides of Teshekpuk Lake due to the high use of these areas by the TCH. We used total rinsed faecal N, C and ash as indicators of diet quality with 1%N identified as the threshold for lean mass gain in female caribou (Barboza *et al.*, 2024). This sampling was done to assess spatial variation of diet quality within one growing season after green-up had occurred. We also assessed landscape attributes to further evaluate spatial variability. To test hypothesis 2, we captured migratory caribou to compare indices of body condition in relation to temporal and spatial variation of green-up over the three years of caribou capture. We used the natural variation in year-to-year phenology to measure body condition in relation to phenology. We used sera chemistries, notably albumin and urea N as indices of body condition due to their ability to reflect short-term nutritional status of both protein and energy when animals are at the lowest point in their fat reserves (Cook *et al.*, 2021; Barboza *et al.*, 2024). We also included body mass and size in our evaluation of condition. Morphological measurements were evaluated for best-fit as covariates of body mass. Body condition indices were paired with faecal samples to establish recent diet quality. We used δ^13^C and δ^15^N as isotopic markers to track dietary changes from indigested diet items in faeces into body tissues. Timing of captures and landscape attributes of capture locations were again used to assess spatial and temporal variability of plant phenology.

## Materials and methods

All procedures for care, handling and experimentation were approved by the Alaska Department of Fish and Game Division of Wildlife Conservation Animal Care and Use Committee (protocol #0035-2023-007, #0035-2024-023, and #0035-2025-020) and Texas A&M AgriLife Research Institutional Animal Care and Use Committee (AUP# 2025-017A).

### Study system

The TCH is one of four migratory herds on the North Slope of Alaska, monitored by the Alaska Department of Fish and Game. The TCH calves in the wetlands around Teshekpuk Lake (70.591637°N, 153.577881°W) while also occupying adjacent areas designated for oil and gas development, including the ConocoPhillips Alaska, Inc. Willow Project area (Fig. 1) (Parrett, 2021; ConocoPhillips, 2025). Annual ranges may span a large portion of the North Slope and into the Brooks Range resulting in overlap with the Western Arctic, Central Arctic and Porcupine caribou herds (Prichard *et al.*, 2022).

**Figure 1:**
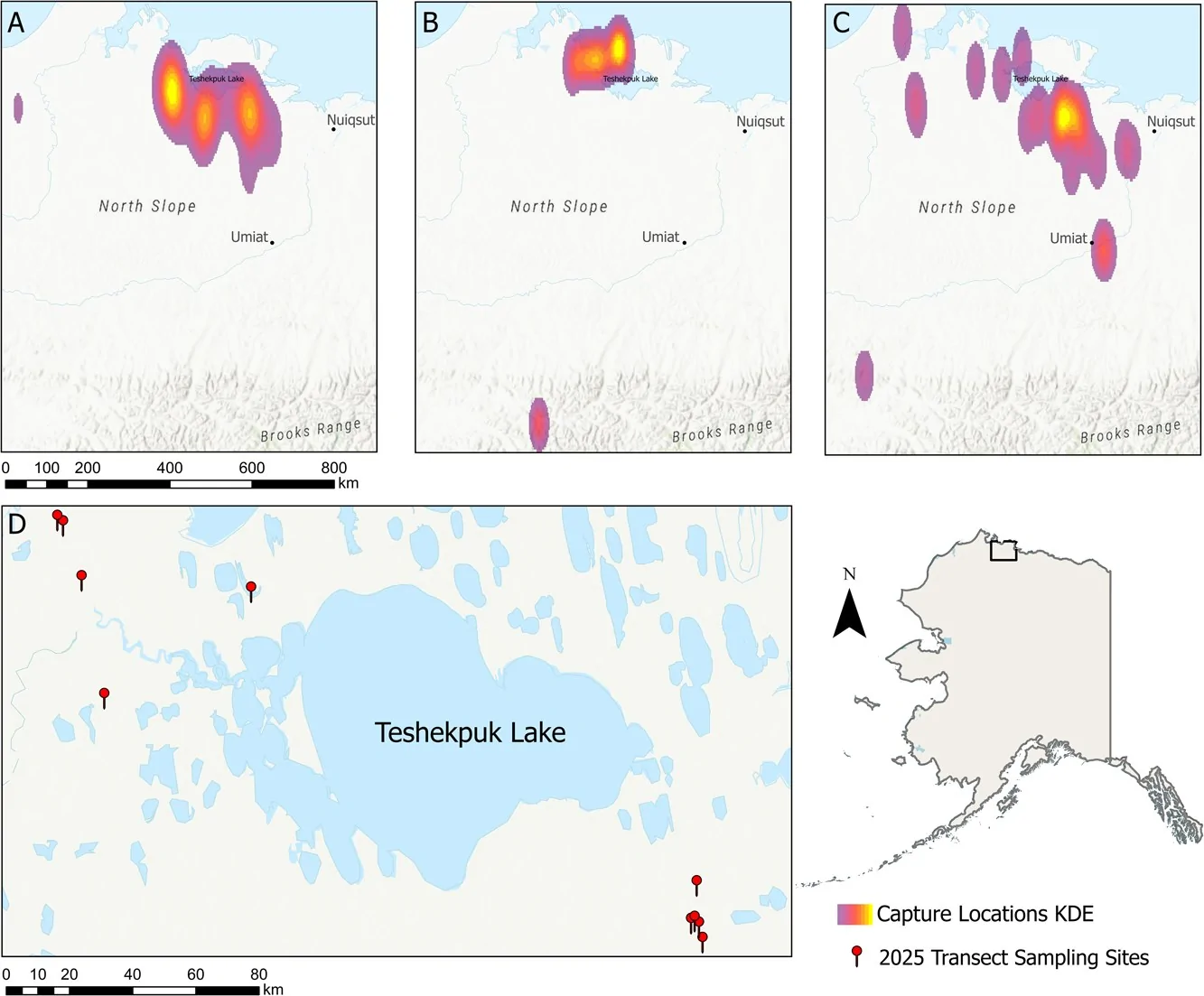
Kernel density estimate of capture locations of caribou in 2022 (**A**), 2023 (**B**) and 2024 (**C**). Transect sampling sites collected in 2025, indicated by pinpoints for sampling faeces in the summer range of the Teshekpuk Caribou Herd (**D**) (Esri, 2025)

### Animal capture

We captured female caribou between 14 and 28 June in three consecutive years from 2022 to 2024 to collect samples, measure body condition and affix GPS collars (Table 1). The period of capture coincides with the post-calving period for the TCH (Carroll *et al.*, 2005; Welch *et al.*, 2022) when movement rates are low and food intakes are also low (Barboza *et al.*, 2024).

**Table 1:** Attributes of sampling sites (*n* = 5) per transect (*n* = 2) in the range of the TCH on 11 and 12 July 2025 with corresponding composition of faeces collected along transects (*n* = 25 samples/eastern transects and *n* = 28 samples/western transect)

	**West of Lake**	**East of Lake**
Median ordinal day	192 ± 1	192 ± 0
Avg latitude (°N)*	70.72426 ± 0.02	70.40598 ± 0.02
Avg longitude (°W)*	154.3016 ± 0.04	152.7937 ± 0.04
Elevation (m)	2.98 ± 1.36^b^	20.07 ± 1.36^a^
Distance to coast (km)	8.83 ± 2.22^b^	26.78 ± 2.22^a^
Cumulative degree days (°C)	252 ± 15.0^b^	331 ± 15.0^a^
Snow cover	0.02 ± 0.00	0.01 ± 0.00
Wind speed (m/s)	7.79 ± 0.29	8.44 ± 0.29
Daily NDVI	0.46 ± 0.01^b^	0.54 ± 0.01^a^
NDVIpre9	0.43 ± 0.01^b^	0.51 ± 0.01^a^
Graminoid cover (%)	31.55 ± 2.25	35.37 ± 2.25
Willow cover (%)	1.60 ± 0.99^b^	5.16 ± 0.99^a^
Lichen cover (%)	0.75 ± 0.89	2.61 ± 0.89
Ash (%DM)	7.03 ± 0.26	7.06 ± 0.26
*N* (crude protein %DM)	22.94 ± 0.50^b^	25.54 ± 0.50^a^
C (%DM)	52.76 ± 0.24^b^	54.57 ± 0.24^a^
δ^15^N (‰)	0.26 ± 0.15^b^	1.63 ± 0.15^a^
δ^13^C (‰)	−29.91 ± 0.05	−29.81 ± 0.05

Values are margins predicted from ANOVA ± standard errors. Letters indicate significant differences among margins within each row (*P* < 0.05).

^*^Latitude and longitude were not included in the ANOVA analysis, raw values reported.

Captures included sub-adults that had not been previously collared as well as adult recaptures for resampling and re-collaring. All sub-adult females were classified as non-reproductive/non-lactating due to lack of calf at heel. We used a small, fixed-wing aircraft to locate groups of caribou that were distributed across their summer range around Teshekpuk Lake (Fig. 1). Recaptured animals were located via telemetry from very high-frequency (VHF) signals from collars. Animals were pursued by an R-44 helicopter for less than five minutes and captured by a net gun without use of chemical immobilization. Restrained animals were promptly blindfolded and hobbled to minimize stress and injury. We estimated the age of newly captured animals based on body size and tooth eruption. We fitted each animal with a global positioning system (GPS) collar before recording body mass and morphometrics. Caribou were weighed (±0.1 kg) using a digital scale and weigh poles. Morphometric measurements included lower jaw length (cm; mandible length to first incisor), ½ heart girth (cm; surface distance from spine to sternum at the distal edge of the scapula) and metatarsal length (cm) (Cattet, 2018). Faeces were collected from the rectum or the ground if voided during restraint. Blood was collected from the jugular, cephalic or saphenous veins into trace element serum polyethylene terephthalate (PET) tubes (BD Vacutainer®, REF 368380, Becton, Dickinson and Company, Franklin Lakes, NJ, USA). Whole blood was allowed to clot at room temperature. We separated serum from the clot by centrifugation at 1500 RCF for 5 minutes. Blood fractions and faeces were stored frozen at −18°C until analysis.

### Faecal transects

To assess diet quality across the summer range, we flew two helicopter transects to locate caribou on 11 to 12 July of 2025. The first transect was on the eastern side of Teshekpuk Lake (152.7736 to 152.8062°W, 70.3846 to 70.4386°N) spanning 58 km long and 65 km wide. The second was on the western side of the lake (154.0572 to 154.6082°W, 70.6161 to 70.7835°N) spanning 18 km long and 3 km wide (Fig. 1). Sampling sites were identified by the presence of three or more caribou, and each transect (one on either side of the lake) had five sampling sites with at least 1 km between each site. After locating groups of animals, we observed their movements and landed to collect freshly voided samples where they were most recently observed. At each sampling site, we collected one to fifteen samples depending on how many animals were present. To minimize resampling of individuals, the number of samples collected did not exceed the estimated number of animals present at each location. Fresh samples were distinguishable from previously deposited material that was frozen and dry.

### Sample processing

Blood clots and faecal samples were freeze-dried for analysis of total N and total C (%), and their respective stable isotopes (δ^15^N and δ^13^C (‰)) at the Alaska Stable Isotope Facility (ASIF, University of Alaska Fairbanks) (Gustine *et al.*, 2014b). Serum proteins were precipitated with sodium tungstate (Barboza *et al.*, 2020) and freeze-dried for isotope analysis. Sera were also analysed for concentrations of creatinine (mg/dL), blood urea N (mg/dL), total protein (g/dL), sodium (mEq/L), potassium (mEq/L) and albumin (g/dL) (dyed with Bromocresol Green (BCG)) using a DxC 700 AU Chemistry Analyser (Beckman Coulter, Brea, CA, USA) at the Texas A&M Preclinical Phenotyping Core (TPPC; RRID: SCR_023263), a facility within the Institute for Genome Sciences and Society at Texas A&M University.

Dried faecal samples were homogenized and milled in a mortar and pestle for determination of analytical dry matter at 80°C and ash content by combustion at 500°C (Barboza *et al.*, 2020). Ground samples were individually rinsed six times in hot water using the methods described by Gustine *et al.* (2014b) to isolate dietary residues from faeces. Rinsed faecal samples were then analysed for N, C and stable isotopes as above at ASIF. Faecal N concentrations are reported as crude protein (Robbins, 2012).

### Landscape attributes

We recorded locations of capture and faecal sampling to a 10-m radius using a GPS unit (Model Aero 660, Garmin, Olathe, KS). Each of the capture (*n* = 110) and transect sampling site locations (*n* = 10) were intersected with raster data to assess landscape attributes at the day of capture or sample collection. Distance to coast (km) was determined using the General Bathymetric Chart of the Oceans (GEBCO, 2024) at a 15 arc-second interval. Elevation (m) was assigned using interferometric synthetic aperture radar (IFSAR) to generate a digital terrain model (DTM) (EROS Center, 2018) aggregated from a 5 to 30 m resolution. NDVI values were derived from MODIS MCD43A4 Version 6.1 at a resolution of 500 × 500 m (Schaaf and Wang, 2021) to measure greenness. We used daily NDVI and the average daily NDVI for the nine days prior to each sample day (NDVIpre9). We used the weather records from ERA5-Land (Muñoz Sabater, 2019) at a resolution of 11 km averaged for each day of collection as follows: air temperature at 2 m above ground (K converted to °C), snow cover as a fraction of the pixel occupied by snow, southern and western wind components (m/s) at 10 m above the ground, and cumulative degree days, which is the cumulative average daily temperature above 0°C from January first to the observation date. Wind speeds were calculated by combining southern and western wind components. Cumulative degree days provide a time weighted measure of plant growing conditions outside of ambient temperature among years. We used estimates of plant functional type top cover with graminoids, willow shrubs and lichen at a resolution of 30 m from Macander *et al.* (2022) to assess available forages for caribou.

### Statistical analyses

All analyses were conducted in StataBE (version 19.5; StataCorp, 2025). We censored data for errors and assessed normality (Shapiro–Wilk) prior to analysis. We used ANOVA to evaluate differences in forage quality and landscape attributes between sampling locations on either side of Teshekpuk Lake. We did not include the average latitude and longitude of transect or capture locations in the ANOVAs due to violation of the assumption of independence of observations. We also used ANOVA with Bonferroni adjustments for pairwise comparisons to assess faecal compositions of captured animals and the attributes of capture locations among years. Five recaptured adults were not included in the ANOVAs for elevation and distance to coast for capture locations as they were in the Brooks Range. We then used ordinary least squares (OLS) regressions to model the variation in faecal N content and faecal stable isotopes of captured animals. Full models include ordinal day, landscape attributes and age groups. Landscape variables were evaluated for correlation using a correlation matrix. Competing models were compared for their fit using Akaike’s Information Criterion (AIC) corrected for small sample sizes.

Caribou that had not been previously captured were estimated to be ~13 months of age and are referred to as sub-adults at time of capture. Two animals captured in 2023 were reclassified as two year olds after evaluating tooth eruption and body mass. All animals two years of age and younger were referred to as sub-adults. Recaptured animals were adults with known records of collar deployment and age. Analyses of captured animal body size and stable isotopes were separated by age groups. We evaluated lower jaw length, ½ heart girth and metatarsal length as covariates for body mass. The body size measurement that had the best fit was used to account for structural size when comparing body mass across years. We used ANOVA and Bonferroni adjustments for pairwise comparisons among years for body mass and body size.

Using methods described by Agostinho *et al.* (2021), we used the Euclidean distance and angle between δ^13^C and δ^15^N tissue values plotted in isotopic space to compare C and N sourcing and recycling from diet and body stores. Isotopic discrimination between serum protein, blood clots and rinsed faeces were calculated for δ^13^C and δ^15^N, respectively, as described by Gustine *et al.* (2014b). We used ANOVA and Bonferroni adjustments for pairwise comparisons among years for Euclidean distances, angles and discrimination factors.

We used ANOVA to compare serum chemistries among age classes and years (Tothova *et al.*, 2018). Creatinine and urea N concentrations were transformed to logarithms to satisfy assumptions of normality for ANOVA. Albumin, creatinine and urea N values were converted to match units used by Milner *et al.* (2003). We used ANOVA and Bonferroni adjustments for pairwise comparisons among years and age classes of data from blood clots and sera.

## Results

### Exp 1. Diet quality and location

We collected 25 faecal samples at 5 different sampling sites on the eastern transect and 28 samples at 5 different sites on the western transect. Within the calving grounds of the TCH, eastern faecal collection sites were at a higher elevation (ANOVA, F1,8 = 79.43, *P* = 0.0000) and farther from the coast (ANOVA, F1,8 = 32.62, *P* = 0.0004) than the western sites (Table 1).

Eastern sites were warmer (cumulative degree days ANOVA, F1,8 = 13.88, *P* = 0.0058), and vegetation cover was dominated by graminoids with significantly more willows on the eastern side (ANOVA, F1,8 = 6.46, *P* = 0.0346) (Table 1). Increased plant growth on the eastern side was indicated by daily NDVI values (ANOVA, F1,8 = 12.05, *P* = 0.0084) and NDVIpre9 (ANOVA, F1,8 = 16.22, *P* = 0.0038) (Table 1).

Caribou diets differed between eastern and western sites within the calving grounds. Faecal ash and analytical dry matter content did not differ between transect locations but faecal N (ANOVA, F1,48 = 13.54, *P* = 0.0006) and faecal C (ANOVA, F1,48 = 27.37, *P* = 0.0000) were highest for the eastern sites (Table 1). Faecal δ13C did not differ between sites, but faecal δ15N was greater at eastern sites (ANOVA, F1,48 = 41.91, *P* = 0.0000) (Table 1).

### Exp 2. Body condition and diet quality

Captures occurred for 3–4 days each year over a 14-day window across years with the earliest captures in 2022 and latest in 2023 (Table 2). We captured 30 sub-adults and 5 adults in 2022, 25 sub-adults and 11 adults in 2023, and 26 sub-adults and 13 adults in 2024. We could not assign reproductive state to adult females because captures occurred one to two weeks after the peak calving period. Although eight adults were lactating and had calves at heel, the remaining 21 animals may have given birth and lost those calves. Not all samples were able to be collected during each capture resulting in variable sample sizes for each analysis. We were able to collect 60 faecal samples and 110 blood samples total. Captures in 2023 occurred at the lowest elevations (ANOVA, F2,102 = 20.45, *P* = 0.0000) and closest to the coast (ANOVA, F2,102 = 42.51, *P* = 0.0000; Table 2). Plant cover types were not significantly different between capture locations among years. However, greenness rates on date of capture and prior to capture were greatest in 2023 (NDVI ANOVA, F2,95 = 14.23, *P* = 0.0000; NDVIpre9 ANOVA, F2,98 = 209.99, *P* = 0.0000; Table 2). Conversely, air temperature (ANOVA, F2,98 = 209.99, *P* = 0.0000) on date of capture was lowest in 2023, but cumulative degree days (ANOVA, F2,107 = 394.14, *P* = 0.0000) was greatest in 2023 (Table 2).

**Table 2:** Attributes of capture locations (*n* = 110) of the TCH from 2022 to 2024 with corresponding composition of faeces collected from captured animals (*n* = 60)

	**2022**	**2023**	**2024**
Ordinal day	165.7 ± 0.14^c^	178.5 ± 0.14^a^	167.5 ± 0.13^b^
Avg latitude (°N)^*^	70.36699 ± 0.10	70.44834 ± 0.10	70.17447 ± 0.10
Avg longitude (°W)^*^	153.755 ± 0.17	154.3045 ± 0.17	153.2354 ± 0.17
Elevation (m)^+^	26.4 ± 4.76^b^	4.40 ± 4.98^c^	47.59 ± 4.57^a^
Distance to coast (km)^+^	40.7 ± 3.16^a^	7.45 ± 3.30^b^	46.27 ± 3.03^a^
Temperature (°C)	7.29 ± 0.44^b^	6.01 ± 0.46^b^	10.84 ± 0.42^a^
Cumulative degree days (°C)	53.6 ± 3.01^c^	160 ± 2.97^a^	64.1 ± 2.85^b^
Snow cover	0.22 ± 0.07	0.00 ± 0.07	0.10 ± 0.06
Wind speed (m/s)	5.52 ± 0.38	5.04 ± 0.40	4.35 ± 0.36
Daily NDVI	0.29 ± 0.03^b^	0.46 ± 0.03^a^	0.24 ± 0.03^b^
NDVIpre9	−0.02 ± 0.02^b^	0.35 ± 0.02^a^	−0.06 ± 0.01^b^
Graminoid cover (%)	22.3 ± 1.72	27.21 ± 1.70	23.83 ± 1.63
Willow cover (%)	4.02 ± 0.46	3.33 ± 0.45	4.72 ± 0.43
Lichen cover (%)	3.45 ± 0.53	2.43 ± 0.52	3.29 ± 0.50
Ash (%DM)	14.5 ± 1.00^a^	8.58 ± 1.00^b^	7.57 ± 1.06^b^
N (crude protein %DM)	5.61 ± 0.56^b^	20.3 ± 0.40^a^	7.20 ± 0.62^b^
C (%DM)	41.9 ± 0.56^c^	53.4 ± 0.40^a^	46.0 ± 0.62^b^
δ^15^N (‰)	−1.10 ± 0.22^c^	0.33 ± 0.16^b^	1.25 ± 0.24^a^
δ^13^C (‰)	−28.4 ± 0.09^a^	−30.4 ± 0.06^b^	−28.6 ± 0.10^a^

Values are margins predicted from ANOVA ± standard errors. Letters indicate significant pairwise differences among margins within each row (*P* < 0.05).

^*^Latitude and longitude were not included in the ANOVA analysis, raw values reported.

+ ^+^Results exclude five adults recaptured in Gates of the Arctic National Park.

Concentrations of faecal N were greatest in 2023 at 3.3% (20.3 crude protein %DM) but only 0.9% (5.61 crude protein %DM) in 2022, which is below the threshold of 1%N (Barboza *et al.*, 2024) (ANOVA, F2,57 = 297.23, *P* = 0.0000; ANOVA, F2,57 = 153.01, *P* = 0.0000) (Table 2).

Conversely, high concentrations of ash and low concentrations of C in faeces indicate the lowest diet quality in 2022. Faecal δ^15^N values were lowest in 2022 but increased by approximately 1‰ each year (Table 2).

We were able to collect 60 faecal samples from caribou captured over three years. Only, 49 of those samples had landscape attribute data that could be associated with their collection locations. Faecal N concentration and δ^15^N were best described by season (DOY) and plant growth (NDVI) confirming the strong phenological effect on diet (Table 3 and Tables  S1 and S2). Faecal δ^15^N was also best described by cumulative degree days and wind speeds. Faecal δ^13^C was best described by season (DOY), distance to coast, elevation (Table 3 and Table  S3). Air temperature was also included in all top models with a significant confidence interval for both faecal N and faecal δ^13^C (Table 3 and Tables  S1 and S3).

**Table 3:** Ordinary least-squares regression models for composition (crude protein (*N*%), δ^15^N and δ^13^C) of faeces collected from captured caribou (*n* = 49)

**Model**		**∆AIC**	**Weight**	***K***	***R*** ^**2**^	**Deviance**
Top	*N*% ~ DOY*, air temperature*, NDVI*, NDVI_pre9_	0	0.5623	4	0.9271	21.8096
Full	*N*% ~ DOY, age group, distance to coast, elevation,	12.96552	0.0009	13	0.9474	5.817516
	air temperature, cumulative degree days, snow cover,					
	wind speed, willow%, graminoid%, lichen%,					
	NDVI, NDVI_pre9_					
Top	δ15N ~ DOY*, cum dd*, NDVI*, NDVI_pre9_*, air	0	0.7898	5	0.3440	126.81316
Full	Temperature δ^15^N ~ DOY, age group, distance to coast, elevation	16.7111	0.0002	13	0.4612	117.17134
	Air temperature, cumulative degree days, snow cover,					
	wind speed, willow%, graminoid%, lichen%					
	NDVI, NDVI_pre9_					
Top	δ^13^C ~ DOY*, distance to coast*, elevation*, air	0	0.5895	5	0.9056	31.80072
Full	Temperature* δ^13^C ~ DOY, age group, distance to coast, elevation	12.54019	0.0011	13	0.9269	15.164436
	Air temperature, cumulative degree days, snow cover,					
	wind speed, willow%, graminoid%, lichen%					
	NDVI, NDVI_pre9_					

Asterisks indicate terms with 85% confidence intervals that do not overlap zero.

We were able to weigh and measure metatarsal length of 80 sub-adults across all three years of capture. Body mass (Y) was directly related to body size measured as jaw length (J), ½ of heart girth (G) and metatarsal length (M) by the following equations (Fig. S1):

*Y* = 2.08J—0.31; *R*^2^ = 0.33; F1,93 = 46.54; *P* < 0.0000.

*Y* = 1.05G—1.47; *R*^2^ = 0.28; F1,93 = 36.16; *P* < 0.0000.

*Y* = 3.63M—78.38; *R*^2^ = 0.43; F1,93 = 70.76; *P* < 0.0000.

We chose metatarsal length as a metric of frame size for sub-adults (Fig. 2). Sub-adult body mass was significantly affected by metatarsal length and year of capture. Sub-adult body mass was 5 kg heavier in 2023 (47.2 ± 0.9 kg) than either 2022 (41.2 ± 0.9 kg) or 2024 (42.0 ± 1.0 kg) after adjusting for frame size (ANOVA, F1,2,76 = 12.44, *P* = 0.0000) (Fig. S2D) (Taillon *et al.*, 2011). Hereafter, body mass refers to values adjusted by metatarsal lengths.

We were able to collect 38 faecal and 81 blood samples from sub-adults across all years to compare isotopically. Among sub-adults, diet from faecal samples was isotopically distinct in all three years with a similar pattern among blood clots albeit with less separation between years (Fig. 3, Table S4, see Fig. S3 for adult plot). δ^15^N values of serum protein were greater than those of blood clots in all years, but δ^13^C values varied with year. Serum proteins followed a similar pattern to diet and clots, but separation between blood fractions varied among years (Table S4).

**Figure 3:**
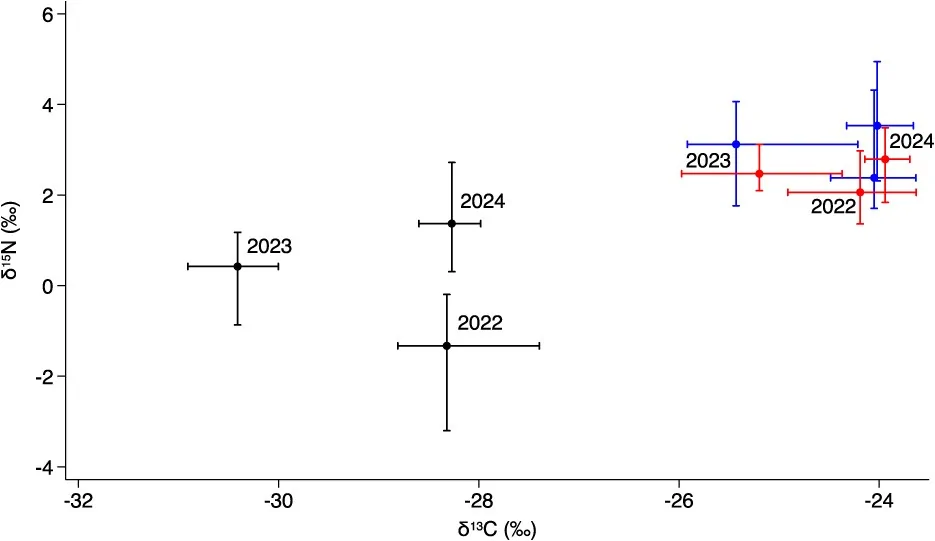
Plotted δ^15^N and δ^13^C isotopic space of rinsed faeces (*n* = 38; black symbols), blood clots (*n* = 81; red symbols) and serum proteins (*n* = 81; blue symbols) of sub-adult captured caribou. Circles denote average values with error bars extending to maximum and minimum values.

Euclidean distances between blood clot (average of previous ~100 days) and serum protein (average of previous ~25 days) did not vary by year for adults (Table S5) but separation between blood fractions for sub-adults was significantly larger in 2023 (1.0‰; Table S4) compared to 2022 ^(^0.6‰) and 2024 (0.8‰) (ANOVA, F2,78 = 8.96, *P* = 0.0003).

Serum chemistries were not different between adults and sub-adults but did vary among years. Serum albumin (44.1 g/L) and urea N (9.9 mmol/L) were highest in 2023 when creatinine concentrations were lowest (69.5 μmol/L) (ANOVA, F2,107 = 8.6, *P* = 0.0003; ANOVA, F2,107 = 28.59, *P* = 0.0000; ANOVA, F2,107 = 50.61, *P* = 0.0000) (Table 4).

**Table 4:** Concentration of protein indices in sera from captured caribou across sampling years (*n* = 110)

	**2022**	**2023**	**2024**
Albumin (g/L)	36.6 ± 1.6^b^	44.1 ± 1.5^a^	36.0 ± 1.5^b^
Creatinine (μmol/L)	120.5 ± 4.9^a^	69.5 ± 4.8^c^	101.1 ± 4.6^b^
Urea *N* (mmol/L)	5.2 ± 0.3^b^	9.9 ± 0.3^a^	5.9 ± 0.3^b^

Values are margins predicted from ANOVA ± standard errors. Letters indicate significant pairwise differences among years within each measure (*P* < 0.05).

Consequently, urea N concentrations were even higher in 2023 (11 mmol/L) after accounting for creatinine concentrations that vary with renal function (ANOVA, F1,2106 = 103.13, *P* = 0.0000).

## Discussion

We supported both of our hypotheses that diet and body condition of arctic migratory caribou respond to variation in plant cover and plant phenology (green-up). We confirmed that spatial variation in plant quality corresponded with changes in dietary N within the calving grounds of the TCH (2025; Table 1). Values of δ^15^N in faeces suggests diets dominated by lichens in 2022, browse in 2023 and graminoids in 2024 (Barboza *et al.*, 2018). We found that serum albumin and urea N were positively associated with onset of green-up in 2023 (NDVI; Table 4), which indicates a positive effect on body protein balance of both adults and sub-adults. Likely as a result of higher quality diet, body mass of sub-adults was also positively associated with onset of green-up.

**Figure 2:**
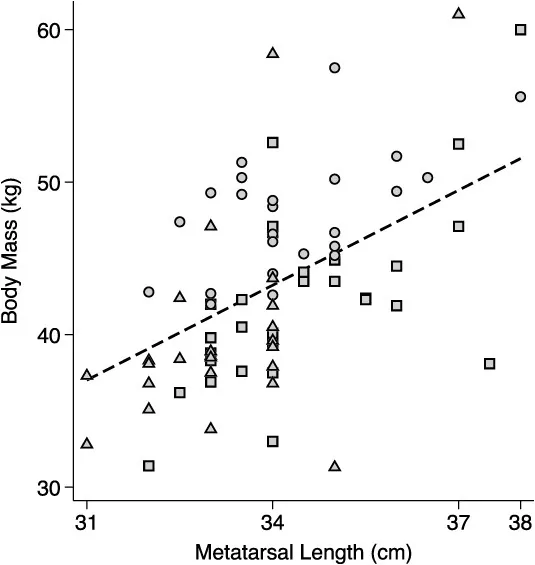
Relationship of sub-adult body mass and metatarsal length (*n* = 80) in 2022 (squares), 2023 (circles) and 2024 (triangles).

Short-term cues from daily foraging returns to energy and nutrient balance would reinforce and refine movements associated with active resource tracking (Stevenson, 2023). Long-term cues of photoperiod integrate physiological responses to life history outcomes such as reproductive onset and success and growth (Arnold *et al.*, 2018). The sensitivity of caribou to shifts in phenological growth and therefore forage quality reflects individual fine-scale responses that therefore drive responses at the level of the herd and the population (i.e. resource tracking) (Gurarie *et al.*, 2024; Tyler *et al.*, 2024; Clark-Wolf *et al.*, 2025). This responsiveness is asymmetrical—selection favours fast gains and slow losses in a system where the window of opportunity to make nutritive gains is short and temporally inconsistent (Barboza *et al.*, 2018; Johnson *et al.*, 2021). Alaskan arctic caribou have demonstrated a robust response to temporal inconsistencies in forage growth (Gustine *et al.*, 2017; Mallory *et al.*, 2020). Arctic caribou are limited by both energy and N, but the window for gaining body protein is earlier and shorter than the window for gaining energy (Barboza *et al.*, 2018). Caribou therefore select for forage quality in early summer when forage levels are suitable for protein gain, but favour forage abundance in late summer for fat gain when forage N declines with senescence (Johnson *et al.*, 2021).

The transition towards high N forages at onset of green-up is evident in the positive trend of faecal δ^15^N from 2022 to 2024, which is consistent with a progression from lichen (low δ^15^N) to browse (intermediate δ^15^N) to graminoids (high δ^15^N) (VanSomeren *et al.*, 2017). At the beginning of green-up, caribou select newly flowered cottongrass, which is rich in digestible N and energy, before switching to browse (Thompson and McCourt, 1981; Jorgenson *et al.*, 2002). Intermediate faecal δ^15^N in 2023 likely indicates caribou had begun selecting browse since green-up had occurred whereas in 2024 high faecal δ^15^N suggests the beginning of green-up (VanSomeren *et al.*, 2017; Hiltunen *et al.*, 2025). Pre-green-up diets were probably similar among years for adult and sub-adult caribou because values for δ^15^N and δ^13^C in red blood cells overlapped (Fig. 3).

Caribou captured in locations with higher NDVI (2023) after the onset of green-up were apparently able to gain body protein quickly as indicated by greater sub-adult body mass, high serum albumin and high urea N. These results are consistent with changes in blood parameters of wild reindeer and caribou from winter to spring (Milner *et al.*, 2003; Johnson *et al.*, 2010; Daleo *et al.*, 2025). The incorporation of dietary protein was also reflected isotopically in the increased Euclidean distances between blood fraction δ^15^N. The enrichment of δ^15^N of serum proteins suggests rebuilding of body stores from an influx of dietary N (Barboza *et al.*, 2020). In this study, high body mass of sub-adult caribou after onset of green-up (2023) was not related to differences in body size, measured as jaw length, ½ of heart girth or metatarsal length. We did not measure body fat but most differences among years in live mass are probably associated with lean mass because caribou do not gain body fat until the later part of summer (Barboza *et al.*, 2024). Caribou can gain lean mass in spring even when body fat is lost (Barboza and Parker, 2008; Barboza *et al.*, 2024). Mass gain has also been reported over winter for both growing and adult caribou (Dale *et al.*, 2008; Couturier *et al.*, 2010; Denryter and Crouse, 2025). We cannot evaluate winter conditions experienced by these animals prior to capture. However, National Weather service weather station data from the Barrow area suggest similar average winter (December to February) temperatures among years (Table S6; Diamond *et al.*, 2013; NOAA, n.d.).

Migratory caribou can move long distances to arrive at foraging grounds where plant growth is likely but fine scale phenological variation (i.e. multiple ‘green waves’) results in variable foraging returns, especially when movement is constrained or increased by endogenous (e.g. parturition), environmental (e.g. deep snow, insects, predators) and anthropogenic (e.g. industrial development) factors (Severson *et al.*, 2021). We found significant variation in dietary N (crude protein) within the calving grounds of the TCH after green-up had occurred (Tables 1 and 2). All samples collected via transect in 2025 and captures in 2023 were above 2%N, while samples collected during captures in 2022 and 2024 were below and at the 1%N threshold of lean mass gains, respectively, (Barboza *et al.*, 2024). Our comparisons among year are consistent with observations of wild cervids harvested throughout the year in which seasonal changes in plant N (green-up) are associated with changes in protein content of the ruminal digesta, kidney mass, liver mass and whole-body mass (Arnold, 2020). Our data confirm that caribou respond to changes within a 14-day window of spring green-up. Further comparisons among age classes, years and herds will be required to refine the sensitivity of caribou to phenological changes in diet quality and quantity.

## Supplementary Material

Web_Material_coag073

## Data Availability

Requests to use the capture location data can be made to the Alaska Department of Fish and Game, as those data are protected under Alaska state statute (AS 16.05.815(d)). All other data will be made available on request.
