# Supplementary material for: A migratory herbivore responds to rapid changes in food supply: diet and body condition of an arctic caribou herd at onset of green-up
Source: Conserv Physiol. 2026 Sep 28;14(1):coag073. doi: 10.1093/conphys/coag073 (PMC13617160; doi:10.1093/conphys/coag073)
Supplement: Supplementary file 1 [file web_material_coag073.zip › Giesen - Supplemental Material (FINAL).pdf]

Supplementary Material
Manuscript Title: A migratory herbivore responds to rapid changes in food supply: diet and body condition of arctic caribou at green-up

Fecal Composition Model Selection

Table S1. Ordinary least squares regression models for total fecal N of feces collected from captured caribou (n = 53). Asterisks indicate terms with 85% confidence intervals that do not overlap zero.

Model	AICc	ΔAICc	Weight	K	R^2	Deviance
N % ~ DOY*, air temperature*, NDVI*, $NDVI_{pre9}$	33.20494	0	0.5623	4	0.9271	21.8096
N % ~ DOY, distance to coast, elevation, air temperature, NDVI, $NDVI_{pre9}$	34.10916	0.90422	0.3578	6	0.9334	32.91474
N % ~ DOY, distance to coast, elevation, air temperature, cumulative degree days, snow cover, wind speed	37.79709	4.59215	0.0566	7	0.9362	18.79709
N % ~ DOY, willow %, graminoid %, lichen %, NDVI, $NDVI_{pre9}$	49.51939	6.44465	0.0224	6	0.9155	17.37745
N % ~ DOY, age group, distance to coast, elevation, air temperature, cumulative degree days, snow cover, wind speed, willow %, graminoid %, lichen %, NDVI, $NDVI_{pre9}$	46.17046	12.96552	0.0009	13	0.9474	5.817516

Table S2. Ordinary least squares regression models for fecal $\delta^{15}N$ of feces collected from captured caribou (n = 53). Asterisks indicate terms with 85% confidence intervals that do not overlap zero.

**Model**	**AICc**	**ΔAICc**	**Weight**	**K**	**R^2**	**Deviance**
$\delta^{15}N$ ~ DOY*, cum dd*, NDVI*, $NDVI_{pre9}$*, air temperature	140.8132	0	0.7898	5	0.3440	126.81316
$\delta^{15}N$ ~ DOY, cum dd, NDVI, $NDVI_{pre9}$, air temperature, wind speed	143.4794	2.6662	0.2082	6	0.3449	126.74772
$\delta^{15}N$ ~ DOY, willow %, graminoid %, lichen %, NDVI, $NDVI_{pre9}$	152.9833	12.1701	0.0018	6	0.2637	136.37866
$\delta^{15}N$ ~ DOY, age group, distance to coast, elevation, air temperature, cumulative degree days, snow cover, wind speed, willow %, graminoid %, lichen %, NDVI, $NDVI_{pre9}$	157.5243	16.7111	0.0002	13	0.4612	117.17134
$\delta^{15}N$ ~ DOY, distance to coast, elevation, air temperature, cumulative degree days, wind speed	179.945	39.1318	0.0000	6	0.3021	163.65928

Table S3. Ordinary least squares regression models for fecal $\delta^{13}C$ of feces collected from captured caribou (n = 53). Asterisks indicate terms with 85% confidence intervals that do not overlap zero.

**Model**	**AICc**	**ΔAICc**	**Weight**	**K**	**R^2**	**Deviance**
$\delta^{13}C$ ~ DOY*, distance to coast*, elevation*, air temperature*	42.97719	0	0.5898	4	0.9056	31.80072
$\delta^{13}C$ ~ DOY, distance to coast, elevation, air temperature, NDVI, $NDVI_{pre9}$	44.30633	1.32914	0.3035	6	0.9058	27.57462
$\delta^{13}C$ ~ DOY, air temperature, cum gdd, willow %, graminoid %, lichen %, NDVI, $NDVI_{pre9}$	47.34725	4.37006	0.0663	8	0.9111	24.73186
$\delta^{13}C$ ~ DOY, air temperature, NDVI, $NDVI_{pre9}$	48.39727	5.42008	0.0392	4	0.08858	37.00192
$\delta^{13}C$ ~ DOY, age group, distance to coast, elevation, air temperature, cumulative degree days, snow cover, southern wind speed, western wind speed, willow %, graminoid %, lichen %, NDVI, $NDVI_{pre9}$	55.51738	12.54019	0.0011	13	0.9269	15.164436

Captured Caribou Body Measurements

We captured 110 female caribou between 1 to 15 years of age including 29 adults and 81 subadults: 5 adults and 30 subadults in 2022, 11 adults and 25 subadults in 2023, 13 adults and 26 subadults in 2024. Metatarsal lengths ranged from 26.0 to 39.5cm in adults and 31.0 to 38.0cm in subadults. Lower jaw lengths ranged from 21.0 to 34.0cm in adults and 13.0 to 32.0cm in subadults. ½ heart girths ranged from 47.0 to 64.0cm in adults and 22.0 to 56.0cm in subadults. Body mass of adults ranged from 52.1 to 82.1kg and of subadults from 31.3 to 61.0kg.

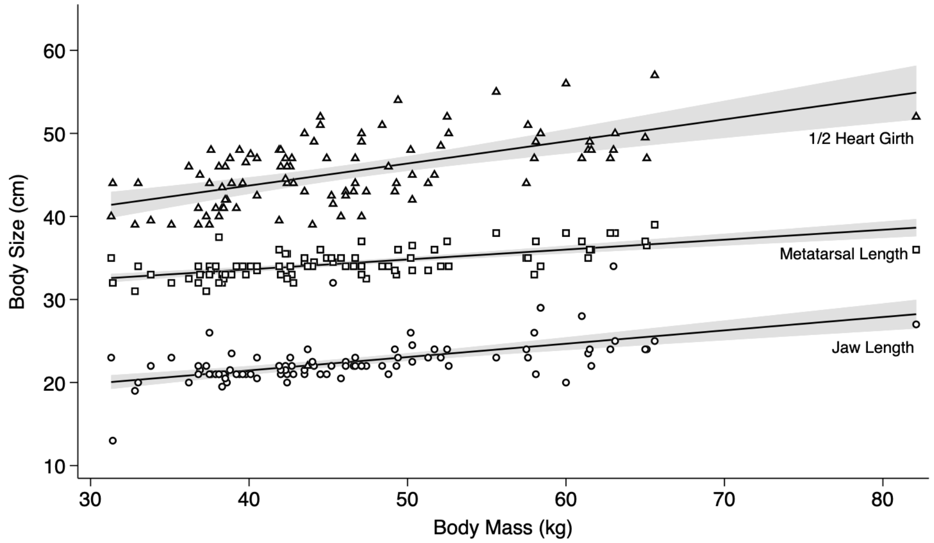

Figure S1. Plotted equations of structural frame size and body mass of captured caribou. Body size measurements include 1/2 heart girth (triangles, n = 107), metatarsal length (squares, n = 108), and jaw length (circles, n = 107).
Alt text: Graph of the linear relationships between body mass and body size denoted by triangles for ½ of heart girth, squared for metatarsal length, and circles for jaw length. All relationship lines have a positive slope.

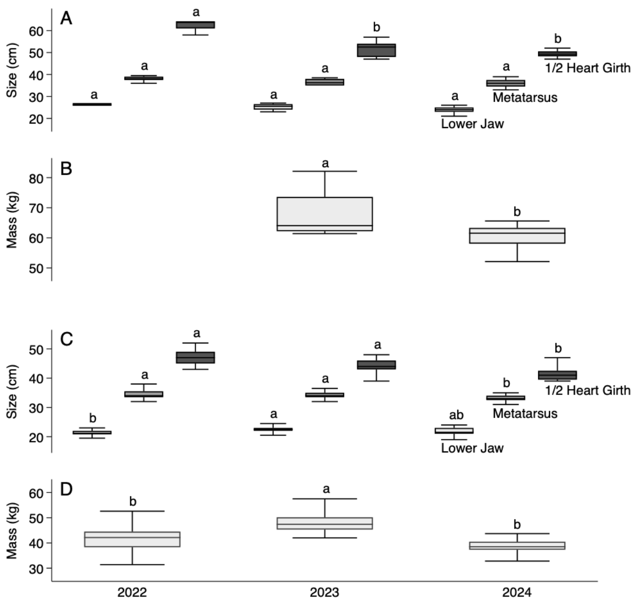

Figure S2. Box plot of adult body size (A; n = 26 for lower jaw and ½ heart girth, n = 27 for metatarsus), adult body mass (B; n = 16), subadult body size (C; n = 81) and subadult body mass (D; n = 80) across years. Body mass values are unadjusted for structural growth. Each box represents 25 percentile, median, and 75 percentile with whiskers at upper and lower adjacent values. Letters indicate significant pairwise differences among means between years ($P < 0.05$). Adults were not weighed in 2022 (B).
Alt text: Box plot of body size and body mass across years with subfigures A and B for adult comparisons and C-D for subadult. Adults were not weighed in 2022. Body size measures includes ½ of heart girth, metatarsus, and lower jaw.

Adult jaw and metatarsus lengths did not vary among years. Adult ½ heart girths were largest in 2022 and not significantly different between 2023 and 2024 (ANOVA, $F_{2,23} = 26.55$, $P = 0.0000$) (Fig S2A). Adult body mass was significantly heavier in 2023 compared to 2024 (ANOVA, $F_{1,12} = 5.00$, $P = 0.0450$) (Fig S2B). Subadults were heaviest in 2023 (Fig S2D) and smallest and lightest in 2024 (Fig S2C and S2D).

Isotope Analysis of Capture Caribou

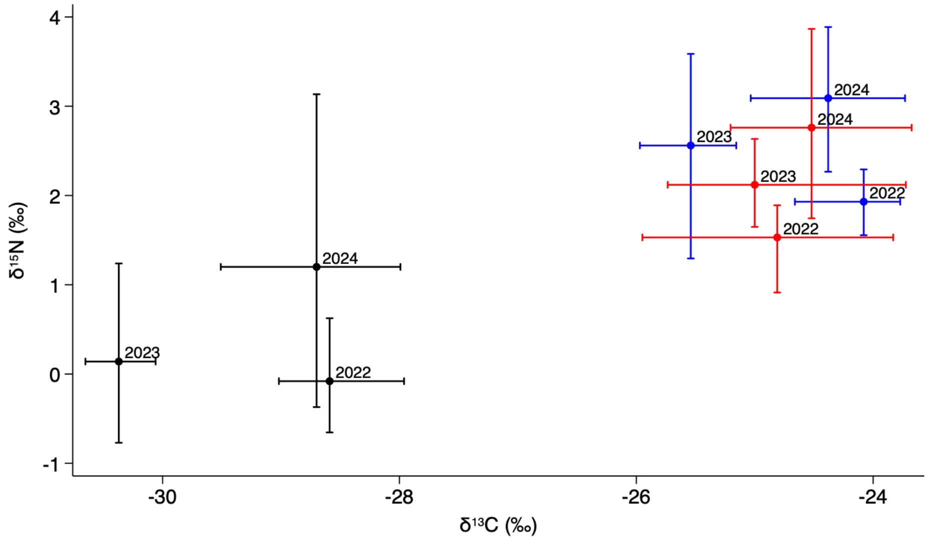

Figure S3. Plotted isotopic space of rinsed feces (n = 22; black symbols), blood clots (n = 29; red symbols), and serum proteins (n = 29; blue symbols) of adult captured caribou. Circles denote average values with error bars extending to maximum and minimum values.

Alt text: Plotted ^{15}N and ^{13}C isotopic values of adult rinsed feces in black, blood clots in red, and serum in blue. Mean values are denoted by circles with error bars extending horizontally and vertically. There are three of each corresponding with each year of collection.

Table S4. Isotope values (mean ± se) of blood clots and serum proteins of subadult captured caribou (n = 81). Letters indicate significant pairwise differences among means within each measure ($P < 0.05$).

	Subadults		
	**2022**	**2023**	**2024**
**Blood Clot**			
$\delta^{15}N$ (‰)	2.06 ± 0.07^{c}	2.47 ± 0.08^{b}	2.79 ± 0.07^{a}
$\delta^{13}C$ (‰)	-24.2 ± 0.05^{b}	-25.2 ± 0.06^{c}	-23.9 ± 0.06^{a}
Euclidean Dist. to Fecal Residue (‰)	5.33 ± 0.18^{ab}	5.66 ± 0.14^{a}	4.62 ± 0.32^{b}
Angle to Fecal Residue (rad)	0.30 ± 0.01^{a}	0.21 ± 0.01^{b}	0.18 ± 0.03^{b}
Discrimination of $\delta^{15}N$ (‰)	3.25 ± 0.22^{a}	2.04 ± 0.17^{b}	1.39 ± 0.40^{b}
Discrimination of $\delta^{13}C$ (‰)	4.19 ± 0.13^{b}	5.25 ± 0.10^{a}	4.33 ± 0.23^{b}
**Serum Protein**			
$\delta^{15}N$ (‰)	2.38 ± 0.11^{b}	3.12 ± 0.12^{a}	3.53 ± 0.12^{a}
$\delta^{13}C$ (‰)	-24.0 ± 0.05^{a}	-25.4 ± 0.06^{b}	-24.0 ± 0.06^{a}
Euclidean Dist. to Fecal Residue (‰)	5.61 ± 0.17^{ab}	5.76 ± 0.14^{a}	4.84 ± 0.31^{b}
Angle to Fecal Residue (rad)	0.30 ± 0.01^{a}	0.24 ± 0.01^{b}	0.23 ± 0.02^{b}
Discrimination of $\delta^{15}N$ (‰)	3.60 ± 0.22^{a}	2.76 ± 0.17^{b}	2.14 ± 0.39^{b}
Discrimination of $\delta^{13}C$ (‰)	4.27 ± 0.13^{b}	5.02 ± 0.10^{a}	4.23 ± 0.23^{b}
**Blood Clot to Serum Protein**			
Euclidean Distance (‰)	0.61 ± 0.07^{b}	1.04 ± 0.08^{a}	0.76 ± 0.07^{b}
Angle (rad)	2.94 ± 3.36^{a}	-0.89 ± 3.68^{a}	-1.16 ± 3.61^{a}
Discrimination of $\delta^{15}N$ (‰)	0.31 ± 0.10^{b}	0.65 ± 0.11^{ab}	0.74 ± 0.11^{a}
Discrimination of $\delta^{13}C$ (‰)	0.14 ± 0.07^{a}	-0.23 ± 0.08^{b}	-0.09 ± 0.08^{ab}

Table S5. Isotope values (mean ± se) of blood clots and serum proteins of adult captured caribou (n = 29). Letters indicate significant pairwise differences among means within each measure (P < 0.05).

	Adults		
	**2022**	**2023**	**2024**
**Blood Clot**			
$\delta^{15}N$ (‰)	1.53±0.23[b]	2.12±0.16[b]	2.76±0.14[a]
$\delta^{13}C$ (‰)	-24.8±0.30[a]	-25.0±0.20[a]	-24.5±0.18[a]
Euclidean Dist. to Fecal Residue (‰)	4.56±0.47[ab]	5.84±0.19[a]	4.71±0.21[b]
Angle to Fecal Residue (rad)	0.23±0.06[a]	0.20±0.03[a]	0.18±0.03[a]
Discrimination of $\delta^{15}N$ (‰)	1.54±0.60[a]	1.96±0.33[a]	1.54±0.34[a]
Discrimination of $\delta^{13}C$ (‰)	4.28±0.43[ab]	5.44±0.23[a]	4.32±0.25[b]
**Serum Protein**			
$\delta^{15}N$ (‰)	1.93±0.26[b]	2.56±0.18[ab]	3.09±0.16[a]
$\delta^{13}C$ (‰)	-24.1±0.16[a]	-25.5±0.11[b]	-24.4±0.10[a]
Euclidean Dist. to Fecal Residue (‰)	4.98±0.39[a]	5.50±0.21[a]	4.87±0.22[a]
Angle to Fecal Residue (rad)	0.23±0.04[a]	0.24±0.02[a]	0.22±0.02[a]
Discrimination of $\delta^{15}N$ (‰)	1.84±0.60[a]	2.49±0.33[a]	2.00±0.35[a]
Discrimination of $\delta^{13}C$ (‰)	4.60±0.25[ab]	4.87±0.14[a]	4.29±0.15[b]
**Blood Clot to Serum Protein**			
Euclidean Distance (‰)	0.91±0.23[a]	1.10±0.16[a]	0.68±0.15[a]
Angle (rad)	-2.22±1.37[a]	0.03±0.93[a]	-1.24±0.85[a]
Discrimination of $\delta^{15}N$ (‰)	0.40±0.24[a]	0.44±0.16[a]	0.33±0.15[a]
Discrimination of $\delta^{13}C$ (‰)	0.72±0.31[a]	-0.54±0.21[b]	0.14±0.19[ab]

Table S6. Summary table of winter conditions for the period of December to February before capture. Data collected from the National Centers for Environmental Information (NOAA).

Winter Period	Capture Year Affected	Average Temp (°C)	Maximum Temp (°C)	Minimum Temp (°C)	Total Sum of Precipitation (mm)
December 2021 to February 2022	2022	-25.2	-4.1	-38.5	19.9
December 2022 to February 2023	2023	-21.0	-3.5	-34.7	20.3
December 2023 to February 2024	2024	-20.3	-3.6	-34.5	31.6
